# Efficacy of prenatal ultrasonography in diagnosing urogenital developmental anomalies in newborns

**DOI:** 10.1186/1471-2393-14-82

**Published:** 2014-02-24

**Authors:** Artur Beke, Fanni Rebeka Eros, Barbara Pete, Istvan Szabo, Eva Gorbe, Janos Rigo

**Affiliations:** 1 1st Department of Obstetrics and Gynecology, Semmelweis University, Baross u. 27., 1088 Budapest, Hungary

**Keywords:** Urogenital developmental disorders, Prenatal sonographic diagnosis, Efficacy of ultrasound

## Abstract

**Background:**

Showing a prevalence rate of 0.5-0.8%, urogenital malformations discovered in newborns is regarded relatively common. The aim of this study is to examine the efficacy of ultrasound diagnostics in detecting developmental disorders in the urogenital system.

**Methods:**

We have processed the prenatal sonographic and postnatal clinical details of 175 urogenital abnormalities in 140 newborns delivered with urogenital malformation according to EUROCAT recommendations over a 5-year period between 2006 and 2010. The patients were divided into three groups; Group 1: prenatal sonography and postnatal examinations yielded fully identical results. Group 2: postnatally detected urogenital changes were partially discovered in prenatal investigations. Group 3: prenatal sonography failed to detect the urogenital malformation identified in postnatal examinations. Urogenital changes representing part of certain multiple disorders associated with chromosomal aberration were investigated separately.

**Results:**

Prenatal sonographic diagnosis and postnatal results completely coincided in 45%, i.e. 63/140 of cases in newborns delivered with urogenital developmental disorders. In 34/140 cases (24%), discovery was partial, while in 43/140 patients (31%), no urogenital malformation was detected prenatally. No associated malformations were observed in 108 cases, in 57 of which (53%), the results of prenatal ultrasonography and postnatal examinations showed complete coincidence. Prenatally, urogenital changes were found in 11 patients (10%), whereas no urogenital disorders were diagnosed in 40 cases (37%) by investigations prior to birth. Urogenital disorders were found to represent part of multiple malformations in a total of 28 cases as follows: prenatal diagnosis of urogenital malformation and the findings of postnatal examinations completely coincided in three patients (11%), partial coincidence was found in 22 newborns (79%) and in another three patients (11%), the disorder was not detected prenatally. In four newborns, chromosomal aberration was associated with the urogenital disorder; 45,X karyotype was detected in two patients, trisomy 9 and trisomy 18 were found in one case each.

**Conclusion:**

In approximately half of the cases, postnatally diagnosed abnormalities coincided with the prenatally discovered fetal urogenital developmental disorders. The results have confirmed that ultrasonography plays an important role in diagnosing urogenital malformations but it fails to detect all of the urogenital developmental abnormalities.

## Background

The prevalence a fetal malformation is high. Levi published in 2002 a summary study, including the RADIUS study and the EUROFETUS study. A total of 36 studies 925,675 fetuses were examined and in 18,443 cases, fetal malformations were detected. The incidence of malformations was between 0.3-3.2%, the average prevalence of 2.0%. The sensitivity of ultrasonography in the detection of developmental disorders: 13.3 to 82.4% (average sensitivity: 40.4%, respectively) [1]. The sensitivity of ultrasound is gradually increased. Crane in 1994 published a summary of the results of the RADIUS study (1987–1991), in total, 7575 fetuses were examined, 182 defects were detected, the prevalence of malformations was 2.4%, the sensitivity of ultrasound was 35.7% [2]. The subsequent Grandjean in 1999, summarized the EUROFETUS study (1990–1993), 170 800 cases during pregnancy examination, 3,685 revealed malformations, fetal malformation prevalence of 2.2%, the sensitivity is 64.1%, respectively [3].

Urogenital developmental malformations discovered in newborns are regarded relatively common due to a prevalence rate of 0.5-0.8% according to the literature [4-6]. Several authors have discussed urogenital disorders [7,8] including those of the kidneys [9-13], and examined hydronephrosis prenatally and postnatally [14-21].

The aim of the current study is to examine the efficacy of sonographic diagnosis in newborns with urogenital developmental malformations.

## Methods

We have processed in a prospetive study the prenatal sonographic and postnatal clinical details of 175 urogenital abnormalities in 140 newborns delivered with urogenital malformation at the 1st Department of Obstetrics and Gynaecology over a 5-year period between 2006 and 2010. Our Prenatal diagnostic center, Perinatal center and Ultrasound Laboratory is a referral unit to which pregnant women with suspected fetal abnormality are referred to.

Urogenital changes representing part of certain multiple disorders associated with chromosomal aberration were investigated separately. According to EUROCAT guidelines (European Surveillance of Congenital Anomalies) we included major malformations and excluded minor anomalies [22]. The patients were divided into three groups; Group 1: prenatal sonography and postanatal examinations yielded fully identical results. Group 2: postnatally detected urogenital changes were partially discovered in prenatal investigations. Group 3: prenatal sonography failed to detect the urogenital malformation identified in postnatal examinations. Newborns with two or more major anomalies were classified as multiple malformations.

Among urogenital malformations, we individually investigated cases in which only pyelectasis could be detected with no further anatomical changes that would cause urinary tract obstruction. In order to make comparisons with international data we individually checked cases in which the narrowing or obstruction of the ureters and urethra had been detected. We also examined the detectability of multicystic and polycystic renal dysplasia, renal agenesis, other urinary tract disorders and genital malformations.

Sonographic investigations were performed in the Ultrasound Laboratory of the 1st Department of Obstetrics and Gynaecology using Philips® HD 11XE (Philips Ultrasound) and GE Voluson® 730PRO (GE Medical System Kretztechnik GmbH & Co OHG) and Medison SA9900 ultrasound device (Medison Co. LTD). The investigations were conducted according to recommendations of FMF, and according to the protocol elaborated by the Hungarian Society of Obstetric and Gynaecological Ultrasonography.

Statistical procession included the investigation of sensitivity, specificity, false negative ratio, false positive ratio, positive predictive value and negative predictive value in the individual cases. Calculating significance, we used the Chi-square (χ2) test. A disorder was regarded significant in case p < 0.05 was established.

Our work complies with the principles laid down in the Declaration of Helsinki. The work has been approved by the ethical committee of the Institutional Review Board of 1st Department of Obstetrics and Gynecology and subjects gave informed consent to the work.

## Results

In the five-year period of investigation, 19 602 newborns were delivered in our department, a total of 140 newborns having congenital urogenital malformations among them (0.7%). During that period in our department were 521 terminations of pregnancy because a fetal malformations, total of 49 fetuses have urogenital malformations among them (9.4%). Also during that period there were 55 spontaneous abortions, and 7 of them have urogenital malformations (12.7%).

Altogether, the 140 newborns exhibited 175 malformations, corresponding to a prevalence rate of 0.7%. Table 1 contains the details of newborns delivered with urogenital malformations. At the time of delivery, the mothers’ mean age was 29.2±5.5 years. On average, deliveries took place around about the ‘verge’ of maturity – at a gestational age of 35.2±4.1 weeks – and the mean of birth weights at 2484.7± 1020.9 g was also lower.

**Table 1:** Birth data of newborns with urogenital malformations (n = 175)

**Type of anomalies**		**Maternal age (years)**				**Gestational weeks**		**Birth weight (grams)**	
	**Cases**	**Average**	**SD**	***Min***	***Max***	**Average**	**SD**	**Average**	**SD**
Hydronephrosis	59	29.03	5.08	*17*	*40*	35.64	3.75	2808.68	826.99
Other obstructive	18	28.61	6.04	*17*	*40*	34.72	4.43	2610.91	1116.7
Renal dysplasia - multicystic	17	26.82	6.53	*17*	*41*	35.82	5.03	2426.67	934.84
Renal dysplasia - polycystic	3	29.33	3.06	*26*	*32*	36.00	3.46	2740.00	860.87
Renal agenesis	16	30.56	6.10	*18*	*40*	33.81	5.53	1916.36	1318.54
Other urinary anomalies	22	28.55	5.63	*16*	*41*	35.00	3.59	2186.36	785.64
Male genital anomalies	26	29.56	4.33	*18*	*38*	34.56	3.85	1929.23	928.97
Female genital anomalies	14	32.14	5.57	*21*	*40*	36.36	3.10	2735.56	787.61
**Total**	**175**	**29.21**	**5.50**	***16***	***41***	**35.19**	**4.14**	**2484.71**	**1020.89**

On average, 4.04±3.49 ultrasound tests were performed during pregnancy; 8±5.57 and 5±3.24 prenatal tests were done in babies born with chromosome aberration and multiple malformation, respectively.

Prenatal ultrasonographic diagnosis and postnatal results completely coincided in 45%, i.e. 63/140 cases in newborns delivered with urogenital developmental disorders. In 34/140 cases (24.3%) discovery was partial, while in 43/140 patients (30.7%), no urogenital malformation was detected (Table 2).

**Table 2:** Accuracy of prenatal detection of neonatal urogenital anomalies (n = 140)

		**I. totally discovered**		**II. partially discovered**			**III. not detected**
	**Cases**	**n**	**%**	**n**	**%**	**n**	**%**
Isolated urogenital abnormalities	108	57	52.8%	11	10.2%	40	37.0%
Associated with chromosome abnormalities	4	3	75.0%	1	25.0%	0	0.0%
Part of multiple malformation	28	3	10.7%	22	78.6%	3	10.7%
**Total**	**140**	**63**	**45.0%**	**34**	**24.3%**	**43**	**30.7%**

Of the 140 newborns, 108 were diagnosed with a single urogenital malformation, 4 cases were associated with chromosome aberrations whereas other multiple malformations were detected in 28 babies.

In the 108 newborns in which urogenital malformation was not associated with disorders in any other organ, the results of prenatal ultrasound tests and postnatal examinations completely coincided in 57 babies (52.8%). Urogenital changes diagnosed prenatally showed partial agreement with postnatal findings in 11 newborns (10.2%) whereas prenatal investigation failed to reveal any disorders in 40 patients (37%).

Among the 28 patients with multiple malformation, complete agreement between prenatal and postnatal diagnoses was found in three cases (10.7%) whereas partial agreement was seen in 22 newborns (78.6%). However, no urogenital malformation was discovered prenatally in three cases (10.7%). Two systems were affected in 12 cases of multiple malformations and in 16 cases, the number of affected systems was ≥3. The associated malformations were as follows: disorders of the extremities (11 cases), cardiovascular malformations (9 cases), disorders of the abdomen and abdominal wall (9 cases), craniospinal malformations and those of the facial cranium (6 cases each), other thoracic disorders (5 cases). In two patients, the associated disorder was diagnosed as fetal hydrops.

Trisomy 9 was diagnosed in one of the four newborns delivered with chromosome aberration (47,XY + 9); the newborn exhibited signs of aortic atresia, hare-lips and cleft palate, hypoplastic nails and distal phalanges in the digits as well as unilateral renal agenesis. Trisomy 18 (47,XY + 18) was also detected in one case. In addition to typical facial dysmorphism, the newborn had atrioventricular septal defect (AVSD), single umbilical artery and horseshoe kidney. Turner syndrome (X-monosomy) was diagnosed in two patients, one of them being a case of Turner mosaic syndrome (45,X/46,XX). In the latter patient, unilateral ovarian cysts were also discovered. The other newborn, with the non-mosaic form (45,X), exhibited unilateral multicystic renal dysplasia.

Among the 140 newborns with urogenital malformations, 122, 17 and 1 were delivered after single, twin and triple pregnancies, respectively. Except for two cases, only one newborn was affected by a urogenital disorder in twin and triple pregnancies. Urogenital disorders were found in both fetuses in two pregnancies. In one of the twin deliveries, both newborn boys had hypospadiasis. In the other case, fetus A had atrial septal defect and unilateral pyelectasis, whereas fetus B had pyelectasis and double kidneys on the same side of his body.

The different urogenital disorders were examined separately. Among the 140 newborns, 175 urogenital disorders were detected. In 77 cases, widening of the urinary passages was found and 59 of those cases were affected by hydronephrosis alone. In 18 patients, some other, obstructive anatomical disorders lay in the background of widening. Cystic kidneys were found in 20 cases, in which multicystic and polycystic renal dysplasia were detected in 17 and 3 cases, respectively. In the remaining two cases, a solitary renal cyst was detected. In 16 patients hypoplasia was identified, or the kidney could not be detected, i.e. the latter cases confirmed the absence of a kidney (Table 3). Table 4 shows the sensitivity, specificity and positive and negative predictive values in the detection of different urogenital malformations.

**Table 3:** Accuracy of prenatal detection of neonatal urogenital anomalies (n = 175)

**Type of anomalies**		**I. totally discovered**		**II. partially discovered**		**III. not detected**	
	**Cases**	**n**	**%**	**n**	**%**	**n**	**%**
Hydronephrosis	59	44	74.58%	2	3.39%	13	22.03%
Other obstructive	18	16	88.89%	2	11.11%	0	0.00%
Renal dysplasia - multicystic	17	14	82.35%	1	5.88%	2	11.76%
Renal dysplasia - polycystic	3	3	100.00%	0	0.00%	0	0.00%
Renal agenesis	16	7	43.75%	4	25.00%	5	31.25%
Other urinary anomalies	22	3	13.64%	10	45.45%	9	40.91%
Male genital anomalies	26	0	0.00%	0	0.00%	26	100.00%
Female genital anomalies	14	8	57.14%	1	7.14%	5	35.71%
**Total**	**175**	**95**	**54.29%**	**20**	**11.43%**	**60**	**34.29%**

**Table 4:** Statistical characteristics of prenatal detection of neonatal urogenital abnormalities (N = 175)

**Type of anomalies**		**I. totally discovered**		**II. + III. not discovered**		***Sensitivity***	***Specificity***	***Positive predictive value***	***Negative predictive value***
	**Cases**	**n**	**%**	**n**	**%**				
Hydronephrosis	59	44	74.58%	15	25.42%	*74.58%*	*98.87%*	*17.05%*	*99.92%*
Other obstructive	18	16	88.89%	2	11.11%	*88.89%*	*99.91%*	*47.06%*	*99.99%*
Renal dysplasia - multicystic	17	14	82.35%	3	17.65%	*82.35%*	*99.86%*	*34.15%*	*99.98%*
Renal dysplasia - polycystic	3	3	100.00%	0	0.00%	*100.00%*	*99.87%*	*11.11%*	*100.00%*
Renal agenesis	16	7	43.75%	9	56.25%	*43.75%*	*99.97%*	*58.33%*	*99.95%*
Other urinary anomalies	22	3	13.64%	19	86.36%	*13.64%*	*99.97%*	*37.50%*	*99.90%*
Male genital anomalies	26	0	0.00%	26	100.00%	*0.00%*	*99.99%*	*0.00%*	*99.86%*
Female genital anomalies	14	8	57.14%	6	42.86%	*57.14%*	*99.99%*	*80.00%*	*99.97%*
**Total**	**175**	**95**	**54.29%**	80	**45.71%**	***54.29%***	***98.43%***	***24.30%***	***99.57%***

Table 5 itemizes other urinary tract malformations (22 cases). Duplicate of urinary tract to different degrees were diagnoses in 12 cases. Female genital malformations could be recognized in 57.14% of the fetuses. No prenatal diagnosis of male genital malformations was made. We also include the gestational age at wich diagnosis was first established (Table 6)*.*

**Table 5:** Other urinary anomalies

**Type of anomalies**	**Cases (n)**
Duplicate of urinary tract	12
Duplex kidney	*4*
Pyleon duplex	*7*
Ureter duplex	*2*
Bladder exstrophy	2
Horseshoe kidney	4
Persistence urachus	1
Cystoanalis/cystourethral fistula	1
Other urinary anomalies	2
**Total**	**22**

**Table 6:** Gestational age at wich diagnosis was first established

**Type of anomalies**	**Average week**	**SD**
Hydronephrosis	28.04	6.84
Other obstructive	26.94	6.84
Renal dysplasia - multicystic	26.07	6.26
Renal dysplasia - polycystic	28.67	2.08
Renal agenesis	28.18	7.22
Other urinary anomalies	29.19	6.65
Male genital anomalies	28.00	7.87
Female genital anomalies	29.42	6.78
**Total**	**29.19**	**6.65**

## Discussion

Based on our study, the prevalence of urogenital malformations was 0.73%. Our data is in close correlation with that of Fadda et al. (0.84%) and exceeded that of Levi et al. (0.51%) [4,5].

In more than half of the cases (52.8%) in which urogenital malformation was the only problem, the disorder could be fully detected prenatally (Table 2). The efficacy of detection was much lower (10.7%) in case the disorder was part of a multiple malformation, the explanation being that the disorders in other organ systems interfered with the detectability of mild urogenital malformations.

Based on our investigations, the sensitivity of detecting urogenital malformations prenatally was 54.29% (Table 4), which was in agreement with studies involving larger populations. Fadda et al. processed the data of a period of 25 years and found that among the 42,256 pregnancies in their sample, urogenital disorders were diagnosed postnatally in 356 cases and 196 cases prenatally, corresponding to a sensitivity rate of 55.06% [4]. In a previous study, Levi et al. investigated 16,370 pregnancies and found the sensitivity rate at 66.67%; they detected urogenital disorders in the fetus in 49 out of 84 cases [5]. VanDorsten, however, had different results in a previous study based on a much smaller sample (2,031 pregnancies), in which the sensitivity of the test was 90% (18 out of 20 cases) [6].

Comparing the individual disorders, our results showed partial agreement with the findings in the literature. Fadda et al. could detect hydronephrosis in 65/72 cases (90.3%) prenatally. In our own studies, we considered pyelectasy exceeding 10 mm and the disorder was found in 44/59 cases (74.58%) [4]. In other obstructive urinary tract disorders, the ratio was higher, 16/18 (88.9%). Based on Fadda’s results, it was found to be 46/55 (83.7%) in the fetus. Our team diagnosed renal dysplasia in 14/17 cases (82.35%) prenatally, in contrast with Fadda et al. who detected the disorder in 12/18 cases (66.7%). Polycystic renal dysplasia occurred in three cases in our sample while Fadda et al. reported four patients in their study; the disorders were detected prenatally in both studies.

Based on the above, the detection of renal and other urinary tract disorders could be regarded as successful but genital disorders were found at a much lower rate. Fadda et al. detected genital disorders in 43/102 fetuses (42.2%), while the relevant data were 8/40 cases (20%) in our sample. The detection of all ovarian malformations was a major achievement in recognizing female genital disorders prenatally, but no other female genital malformations or male genital disorders were identified in prenatal investigations.

## Conclusions

Postnatally and prenatally diagnosed fetal urogenital developmental malformations coincided in almost half of the cases. Our results have confirmed that ultrasound tests play an important role in diagnosing urogenital malformations but they do not always allow the detection of all urogenital developmental malformations. It has been concluded that among the disorders such as fetal hydronephrosis and other obstructive uropathies as well as the cases of renal dysplasia and female genital malformations, it is ovarian cysts that can be detected with great certainty in the fetus prenatally. In contrast, other renal disorders and male genital malformations are found at a low rate. Being aware of the above is important for experts performing ultrasound tests, health professionals in providing genetic counselling and prenatal care and, also, neonatologists and paediatricians seeing newborns. During prenatal care, the expectant mother should be given adequate information about the efficacy of the examinations. If a malformation is detected postnatally, the couple should also be informed how reliably the specific malformation is detectable by prenatal sonography.

## Competing interests

The authors declare that they have no competing interests.

## Authors’ contributions

AB and EFR participated in the design of the study and performed the statistical analysis and drafted the manuscript. EFR and BP collected the data from patients. IS performed the ultrasound examinations. EG and BP performed the examination of the newborns at neonatal intensive care unit. RJJ read and approved the final manuscript and participated in the design of the study. All authors read and approved the final manuscript.

## Pre-publication history

The pre-publication history for this paper can be accessed here:

http://www.biomedcentral.com/1471-2393/14/82/prepub
